# Interaction between the Natural Components in Danhong Injection (DHI) with Serum Albumin (SA) and the Influence of the Coexisting Multi-Components on the SaB-BSA Binding System: Fluorescence and Molecular Docking Studies

**DOI:** 10.1371/journal.pone.0128919

**Published:** 2015-06-02

**Authors:** Jia Hao, Yingyue Zhang, Xingrui Wang, Huo Yan, Erwei Liu, Xiumei Gao

**Affiliations:** 1 Tianjin State Key Laboratory of Modern Chinese Medicine, Tianjin University of Traditional Chinese Medicine, Tianjin, 300193, PR China; 2 Tianjin Key Laboratory of Phytochemistry and Pharmaceutical Analysis, Tianjin University of Traditional Chinese Medicine, Tianjin, 300193, PR China; CNR, ITALY

## Abstract

Danhong injection (DHI) is a widely used Chinese Materia Medica standardized product for the clinical treatment of ischemic encephalopathy and coronary heart disease. The bindings of eight natural components in DHI between bovine serum albumin (BSA) were studied by fluorescence spectroscopy technology and molecular docking. According to the results, the quenching process of salvianolic acid B and hydroxysafflor yellow A was a static quenching procedure through the analysis of quenching data by the Stern-Volmer equation, the modified Stern-Volmer equation, and the modified Scatchard equation. Meanwhile, syringin (Syr) enhanced the fluorescence of BSA, and the data were analyzed using the Lineweaver-Burk equation. Molecular docking suggested that all of these natural components bind to serum albumin at the site I location. Further competitive experiments of SaB confirmed the result of molecular docking studies duo to the displacement of warfarin by SaB. Base on these studies, we selected SaB as a research target because it presented the strongest binding ability to BSA and investigated the influence of the multi-components coexisting in DHI on the interaction between the components of the SaB-BSA binding system. The participation of these natural components in DHI affected the interaction between the components of the SaB-BSA system. Therefore, when DHI is used in mammals, SaB is released from serum albumin more quickly than it is used alone. This work would provide a new experiment basis for revealing the scientific principle of compatibility for Traditional Chinese Medicine.

## Introduction

**Fig 1:**
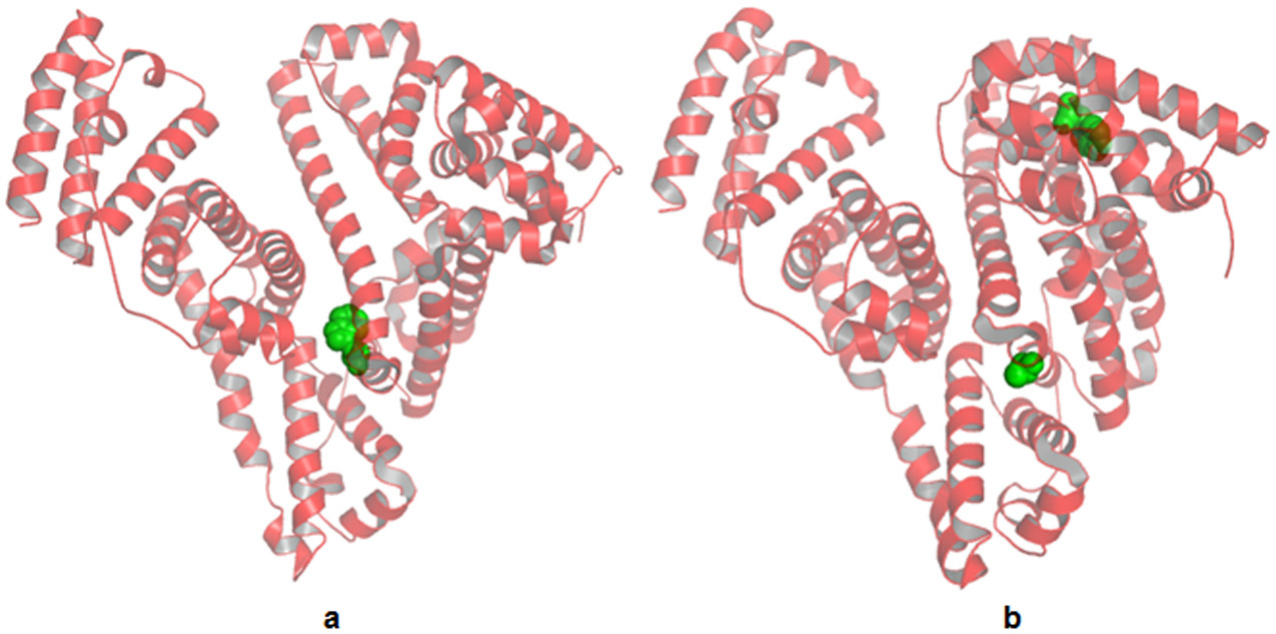
Chemical structures of HSAand BSA.

Human serum albumin (HSA, Fig 1a) is the most abundant plasma carrier protein in humans and is responsible for transporting many endogenous and exogenous agents [1, 2]. The drug binding ability of HSA is directly related to the effectiveness of clinical therapy due to its strongly influences on the free drug concentrations in the plasma. Therefore, drug-binding ability towards HSA is a crucial factor that should be carefully considered in drug research [3]. Bovine serum albumin (BSA, Fig 1b) has an excellent structural similarity with HSA and a repeating pattern of disulphide bonds [4]. So it has been studied extensively as a replacement for HSA due to its easy accessibility and high stability [5–9].

**Fig 2:**
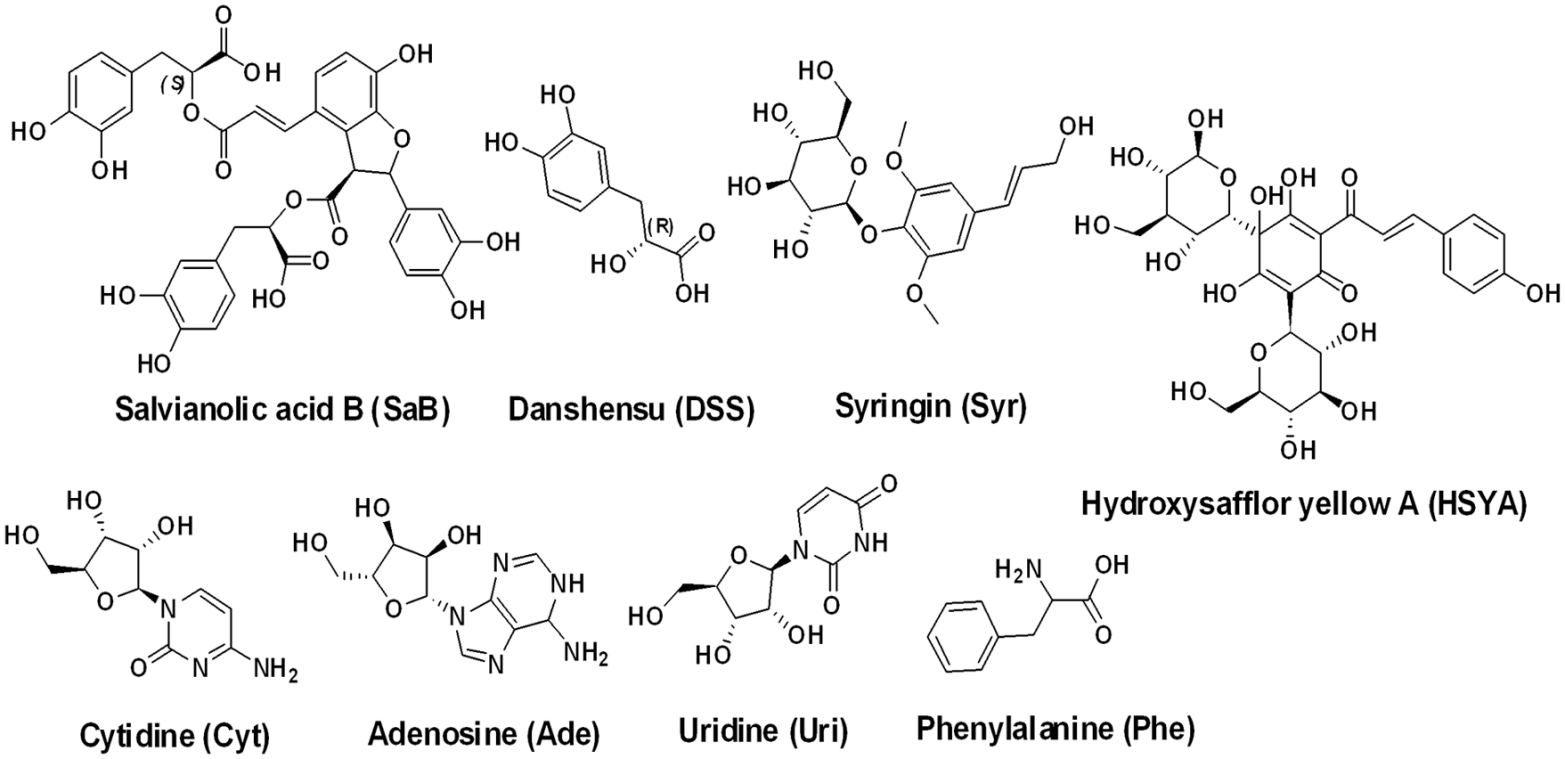
Chemical structures of active components in DHI.

Danhong injection (DHI) is a Chinese Materia Medica standardized product that is extracted from Danshen (Radix Salviae miltiorrhizae) and Honghua (Flos Carthami tinctorii). DHI is mainly used to clinically treat ischemic encephalopathy and coronary heart disease due to its ability to promote blood circulation and resolve stasis to promote regeneration[10, 11]. Previous studies demonstrated that salvianolic acids from Danshen (Radix Salviae Miltiorrhizae), and nucleosides, flavonoids, phenylethanoid glycosides from Honghua (Flos Carthami Tinctorii) might be the most potent active components for the therapeutic effect of DHI[12–15]. In vitro experiments have identified some of these natural components binding to HSA or BSA [16–19],but many components still need to be carefully studied. These studies were carried out under relatively “pure” conditions, ignoring the complex conditions under which natural products exist in plants. Indeed, as in DHI, a variety of main components coexist together, affecting the interaction between a single component with serum albumin. Therefore, investigating these active components’ (Fig 2) binding abilities of BSA and discussing the influence on serum albumin binding between these components when coexisting would provide meaningful insight into the transport, distribution, metabolism of DHI, and a new perspective to reveal the compatibility principle of DHI.

Fluorescence spectroscopy technology has been widely used to investigate the interactions of drug protein system [20–24]. Generally, the fluorescence spectrum of BSA is dominated by tryptophan residues (Trp-212 and Trp-134, Fig 1). Because of the high sensitivity of these two tryptophan residues to the local environment, we use the intrinsic tryptophan fluorescence as a probe to study the interactions between the natural components in DHI and serum protein.

Molecular docking is an important technique for computer-aided drug design (CADD) [25–27]. This technique could provide the detailed orientation of protein-drug system including the binding mode and the structural conformation of the drug molecule.

In this paper, the binding between these active components and BSA was subsequently investigated by a fluorescence technique. The binding parameters K_a_, K_b_, K_sv_, K_q_and n were thus determined. The molecular docking between these components and BSA/HSA were discussed using AutoDock Vina software to identify the binding site of each compound. Moreover, we explored the influence of the multi-components coexisting in DHI on the interaction of single component with BSA.

## Materials and Methods

### 2.1. Materials

Bovine serum albumin (BSA) was obtained from Solarbio China and was used without further purification. Salvianolic acid B (SaB), adenosine (Ade), cytidine (Cyt), uridine (Uri), phenylalanine (Phe), warfarin (War), and ibuprofen (Ibu) were obtained from the National Institute for Food and Drug Control (Beijing, China). Hydroxysafflor yellow A (HSYA), syringin (Syr) and danshensu (DSS) were purchased from Zhongxin Innova Laboratories (Tianjin, China). The SaB-free DHI sample was obtained from WuXi AppTec (Tianjin) Co., Ltd. All of the other chemicals were used as supplied without further purification.

### 2.2. Preparation of the Stock Solutions

BSA stock solution (0.1 mM) was prepared in a physiological aqueous solution with 100 mM phosphate buffer, pH 7.2. The stock solution of warfarin (10 mM) was prepared in methanol. The preparation of ibuprofen and the tested components stock solutions (0.1 mM) were similar with BSA stock solution. All of the stock solutions were kept at 4°C in the dark.

### 2.3. Fluorescence Spectroscopy Studies

All of the fluorometric experiments were recorded on a multifunctional microplate reader (Perkin-Elmer Corporation USA). The stock solutions of BSA and the tested components were diluted to the required experimental sample concentrations and mixed in multiple colorimetric tubes. For SaB and SYR, the range of concentrations was 0, 2.5, 5, 10, 15, 20, 25, 30, 35, 40 μM from 1 to 10; for HSYR, the range of concentrations was 0, 25, 50, 60, 70, 80, 90, 100 μM from 1 to 8; for DSS, CYT, URI, ADE, and Phe, the range of concentrations was 0, 20, 40, 50, 60, 70, 90, 100 μM from 1 to 8. The test solutions for fluorescence spectroscopy were prepared with different concentrations via stepwise dilution. To avoid the inner filter effect, we used very dilute solutions in the experiment, ensuring a final concentration of BSA of 5 μM. The test solutions were incubated at 310 K for 40 min and then transferred into 96-well black fluoro-microplates (CORNING, USA); the fluorescence spectroscopy tests were carried out at the same temperature to simulate the body temperature. The excitation wavelength was 280 nm, and the emission spectra were recorded from 300 to 500 nm. The maximum emission intensities were used to calculate binding parameters. In order to correct the background, appropriate blanks were subtracted according to series preliminary experiment.

### 2.4. Molecular Docking

The three-dimensional (3D) crystal structures of BSA (entry code 4JK4) [28] and HSA in complex with warfarin (entry code 1H9Z) [29] and with ibuprofen (entry code 2BXG) [30] were obtained from the Protein Data Bank (PDB). The two dimensional (2D) structures of tested components were drawn using ChemBioDraw Ultra, and then converted to three-dimensional (3D) structures by ChemBio 3D Ultra. The initial three-dimensional structures were optimized using the MMFF94 force field with default parameters. Then the Auto Dock Tools (ADT) soft was used to convert the mol2 files into the final coordinate files for docking studies. A molecular docking simulation of those compounds was performed with the AutoDock Vina program. Each computation was performed using the default parameters as described in the AutoDock Vina manual except for grid dimensions. Considering the differences of molecule size, each grid computation was performed as large as possible with a grid box of 30×30×30 Å, with 1 Å spacing, to ensure that there were enough box dimensions to cover all the active site residues and to allow for the flexible rotations of all molecules. And then, the grid was centered at the middle of Sudlow's site I and Sudlow's site II, respectively. The docking result was shown by PyMOL.

## Results and Discussion

### 3.1. Fluorescence spectrometry of a single component with BSA

**Fig 3:**
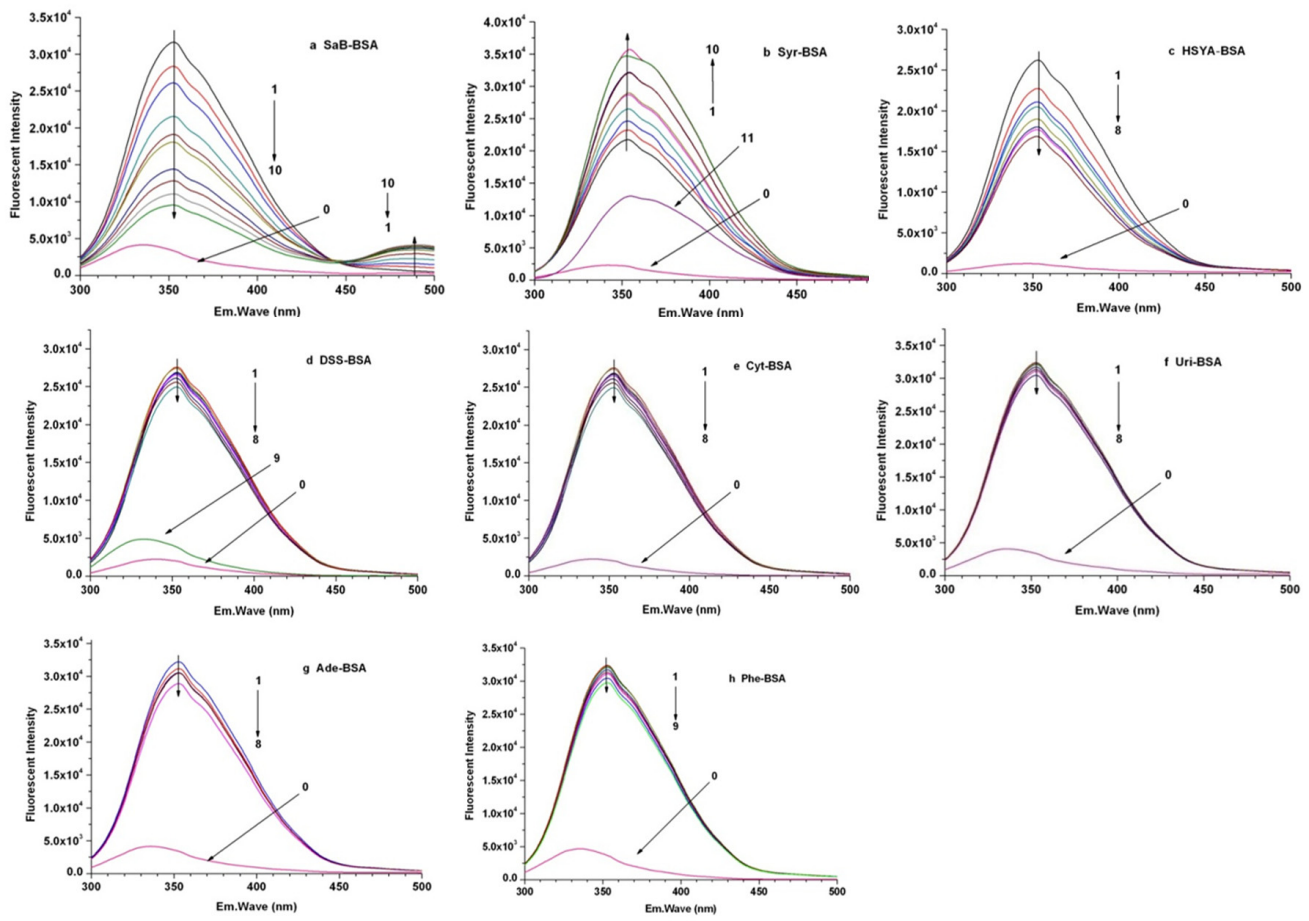
Fluorescence spectra of BSAin absence and presence of different tested components.

The binding of the tested natural components would cause different changes in the fluorescence spectra of BSA due to the protein conformation transformation. The interactions between the tested components and BSA were studied by analysis of the fluorescence spectra of BSA in the absence and presence of different concentration of tested components. The effect of these components on the BSA fluorescence spectra is as shown in Fig 3.

As shown in Fig 3, when the excitation wavelength was fixed at 280 nm, BSA had a strong fluorescence emission band at 355 nm. The decrease of the fluorescence intensity value of BSA at 355 nm followed with the successive addition of SaB and HSYA indicated that these two components could quench the fluorescence of BSA. Meanwhile, the increase of the fluorescence intensity value of BSA at 355 nm followed with the successive addition of Syr indicated that Syr could enhance the fluorescence of BSA. All these results suggested that SaB, HSYA, and Syr could bind to BSA and induce the microenvironment transformations around the BSA protein. The peak position of each fluorescence emission spectra was maintained at 355 nm without any blue shift or red shift, suggesting that the binding of these three natural components did not cause obvious changes in the polarity environment around the chromophore of tryptophan [31, 32]. The quenching efficiency of SaB was much greater than that of HSYA at the same concentration, and the subsequent addition of SaB increased the emission at ~490 nm due to SaB being bound to BSA. In addition, the occurrence of an isoactinic point at 445 nm was considered as direct evidence of the existence of SaB-BSA complex, which suggested that there to be a static quenching process in the SaB-BSA binding system. Beyond these three components, there was no obvious change in the fluorescence spectra of BSA with the successive addition of the other five components, possibly due to the very low binding of these five components with BSA leading to very little changes in the microenvironment around the chromophore of tryptophan.

### 3.2. Fluorescence parameter analysis

**Fig 4:**
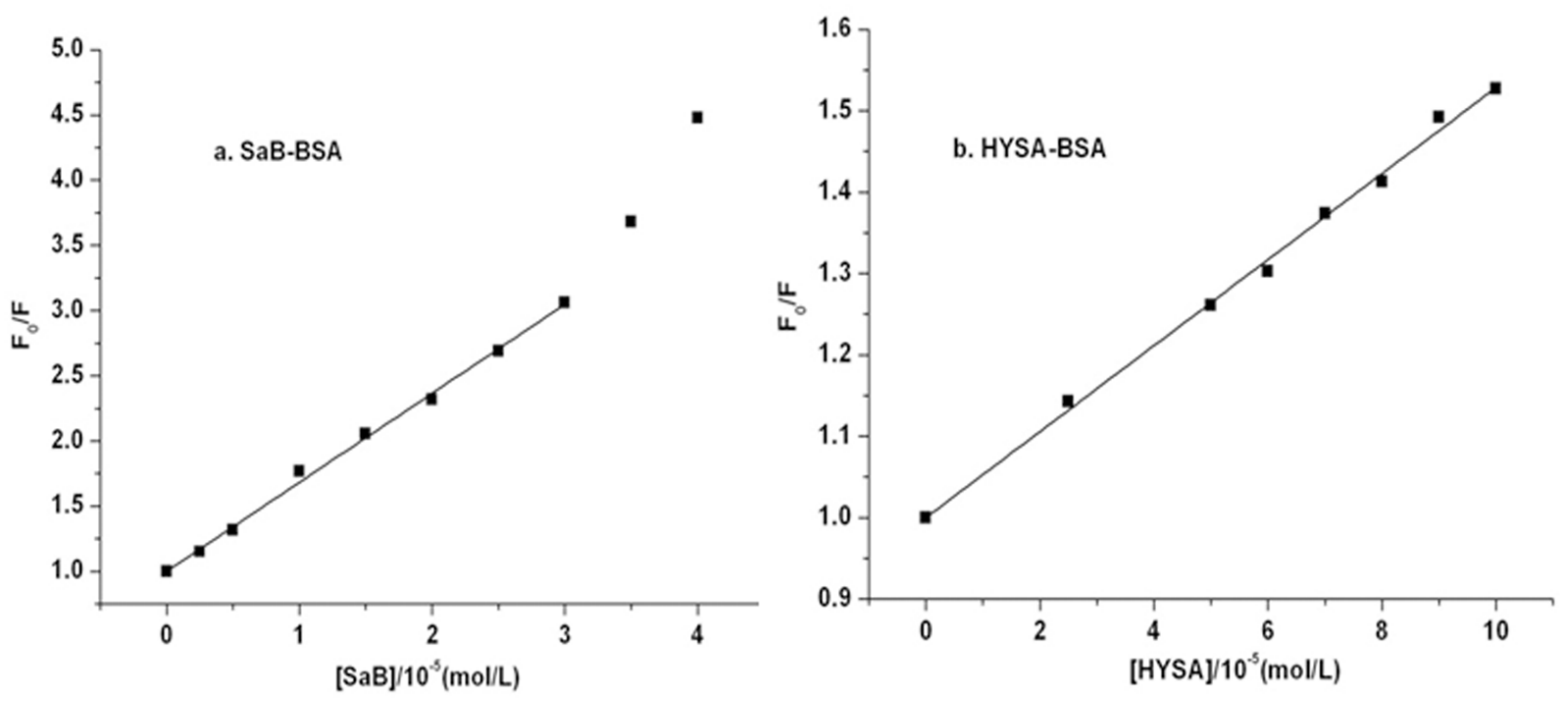
Stern-Volmer plots of SaB-BSA system and HYSA-BSA system.

**Fig 5:**
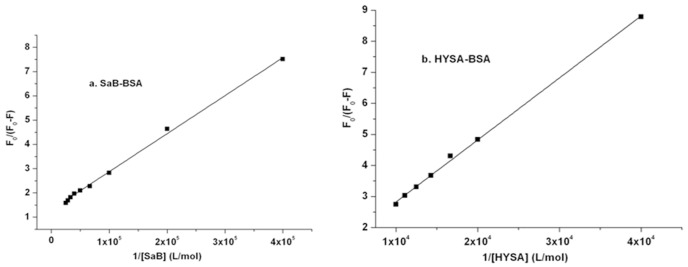
Modified Stern-Volmer plots of SaB-BSA system andHYSA-BSA system.

**Fig 6:**
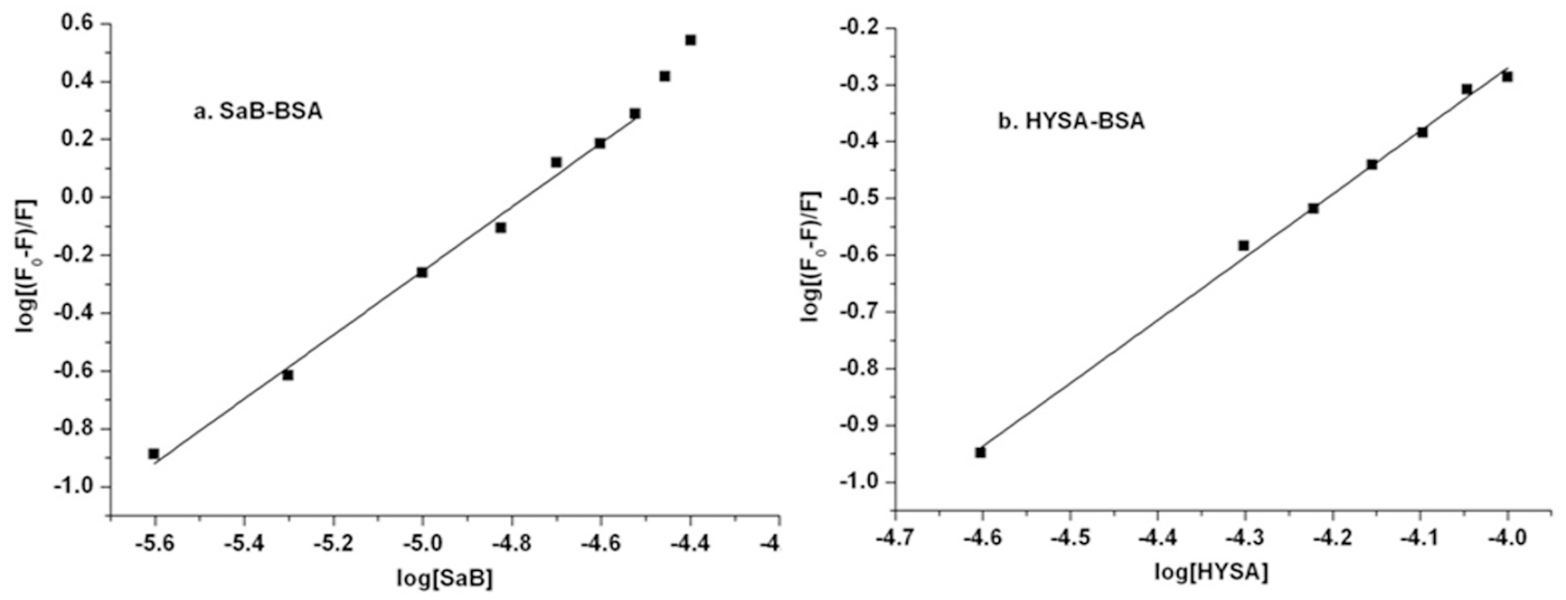
Modified Scatchard plos of SaB-BSA system and HYSA-BSA system.

To illustrate the binding profiles of these tested components, the fluorescence emission intensity data for BSA at the peak position were analyzed by the followed equations ((Eq 1)–(Eq 4)), as shown in Figs 4–6. According to fluorescence spectrometry studies, SaB and HSYA can quench the intrinsic fluorescence of BSA, and the quantum yield of fluorescence decreased with the increasing quencher molecule concentration. Fluorescence quenching can be divided into static quenching procedure and dynamic quenching procedure. The static quenching is usually resulted from the formation of a stable complex between the protein and quencher, while, the dynamic quenching is usually resulted from collisional encounters between the protein and quencher [33]. Both the static and dynamic quenching procedure can be described using the linear Stern-Volmer equation[34–38] as follows:

$$F_{0}/F\;=\;1+K_{q}\tau_{0}\left[Q\right]\;=\;1+K_{sv}\left[Q\right]$$ (1)

As shown in Fig 4, the curves of F_0_/F versus [Q] had a good linearity when the ratio of C_SaB_/C_BSA_ ranged from 0.5 to 6 and when the ratio of C_HYSA_/C_BSA_ ranged from 5 to 20, which were plotted according to Eq (1). When the ratio of C_SaB_/C_BSA_ was larger than 6, the Stern-Volmer plot of the SaB-BSA system changed to an upward curvature, indicating a combined quenching procedure at a higher concentration ratio of C_SaB_/C_BSA_.

The modified Stern-Volmer equation (Eq (2)) is another way to describe the quenching process[33, 39]. The modified Stern-Volmer plots of the SaB-BSA system and the HYSA-BSA system are shown in Fig 5.

$$F_{0}/\Delta F\;=\;F_{0}/(F_{0}-F)\;=\;1/f_{a}+1/(f_{a}K_{a}\left[Q\right])$$ (2)

The fluorescence emission intensities of BSA can also be analyzed the modified Scatchard equation as follows:

$$log[\left(F_{0}-F\right)/F]\;=\;logK_{b}+nlog\left[Q\right]\;$$ (3)

Though the modified Scatchard equation, log[(F_0_-F)/F] was plotted against log[Q], and the values of K_b_ and n were found from the intercept and slope, respectively, as shown in Fig 6.

**Fig 7:**
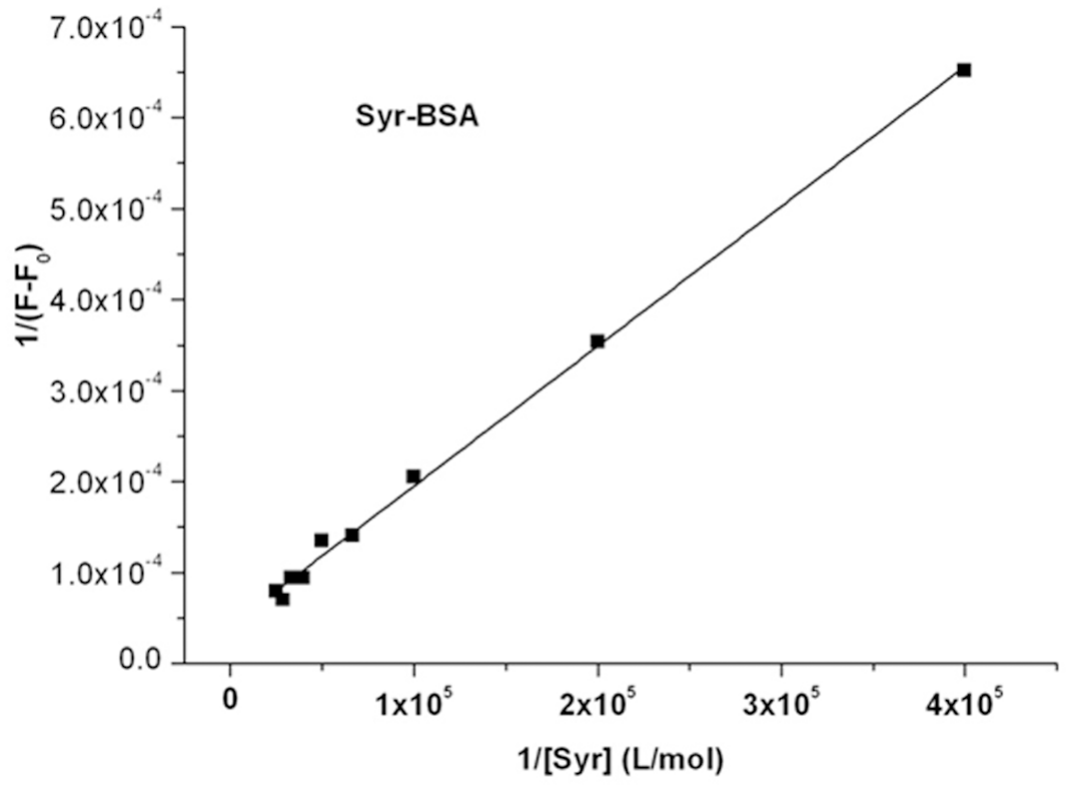
Lineweaer-Burk plots of SYR-BSA system.

However, the intensity of BSA intrinsic fluorescence increased with the increasing Syr concentration. In this condition, the fluorescence data were analyzed by the Lineweaver-Burk equation as follows [40, 41], and the result was shown in Fig 7.

$$1/\Delta F\;=\;1/\Delta F_{max}+\Delta F_{max}/K\left[Q\right]\;=\;1/\left(F_{\infty }-F_{0}\right)+\left(F_{\infty }-F_{0}\right)/K\left[Q\right]\;$$ (4)

The calculated fluorescence parameters of a single component binding with BSA are presented in Table 1. In our study, the binding parameter K_q_ of SaB which was calculated by the Stern-Volmer Eq (1) was 6.83×10^12^ M^-1^ s^-1^ and was much larger than that of HSYA (0.53×10^12^ M^-1^ s^-1^). These two calculated K_q_ values were all far higher than the maximum scatter collision quenching constant (2×10^10^ M^-1^ s^-1^). It is indicated that both of the quenching processes of SaB and HSYA to BSA were initiated by static quenching mechanism. This result confirmed the above analysis of fluorescence emission spectrometry. The binding parameters K_a_ that were obtained from the Eq (2) for SaB and HSYA were 8.34×10^4^ M^-1^ and 0.31×10^4^ M^-1^, respectively. The values of K_b_ and n of SaB and HSYA were also calculated by plotting log[(F_0_-F)/F] against log[Q]. The binding constant (K_b_) of SaB (18.6×10^4^ M^-1^) was still larger than that of HSYA (1.48×10^4^ M^-1^). The value of n for SaB and HSYA was 1.10and 1.11, respectively. Therefore, it has been proved that there was only one class of binding site for SaB and HSYA on BSA. All of the parameters for SaB were consistent with previous studies [16]. Based on the above data, we concluded that SaB-BSA binding was stronger than the binding of HSYA-BSA due to the much higher values of K_SV_, K_q_, and K_b_ [42]. As to fluorescence enhancement situation, the fluorescence data of Syr were analyzed using Eq (4). The binding constant K was 2.7×10^4^ M^-1^ for the Syr-BSA system.

**Table 1:** The calculated fluorescence parameters of single tested component with BSA, at 310 K and pH 7.2.

	SaB	HSYA	Syr	Phe	DSS	Uri	Cyt	Ade
K_SV_ (×10^4^ M^-1^)	6.83	0.53	--	--	--	--	--	--
K_q_ (×10^12^ M^-1^s^-1^)	6.83	0.53	--	--	--	--	--	--
K_a_ (×10^4^ M^-1^)	8.34	0.31	--	--	--	--	--	--
K_b_ (×10^4^ M^-1^)	18.6	1.48	--	--	--	--	--	--
n	1.10	1.11	--	--	--	--	--	--
K (×10^4^ M^-1^)	--	--	2.7	--	--	--	--	--

Based on the above studies, SaB is the most highly bound to serum albumin. That means that compared with the overall amount of SaB, the amount of SaB that is available to diffuse into the target tissue may be significantly reduced, and the pharmacological effects may consequently be poor. However, the multi-components coexisting in DHI would indeed affect the binding of SaB-serum albumin system, thereby changing the free SaB concentration in the plasma. Therefore, the influence of the coexisting multi-components on SaB-serum albumin binding should be carefully studied to determine the scientific principle of compatibility for Traditional Chinese Medicine and to further ensure safety in clinical application.

### 3.3. Site marker competitive experiments for SaB

Site marker competitive experiments were carried out to identify the binding site of SaB on BSA using molecules that were well known as specifical binding markers of BSA. BSA is a globular heart-shaped non-glycoprotein, and has two ligand-binding sites located in the hydrophobic cavity, which referred as Sudlow's site I and site II [43]. From X-ray crystallographic studies, warfarin has been demonstrated to be the site marker of site I in HSA, while ibuprofen is a site II binder [2]. Due to the 88% sequence identity between BSA and HSA [44], warfarin and ibuprofen are still used as site markers for site I and site II of BSA, respectively, even if there are no exact X-ray crystal structures for the warfarin-BSA complex or the ibuprofen-BSA complex. Thus, information about the SaB binding site can be obtained by comparing the differences in the fluorescence of the SaB-BSA binding system in the absence and presence of the two markers.

**Fig 8:**
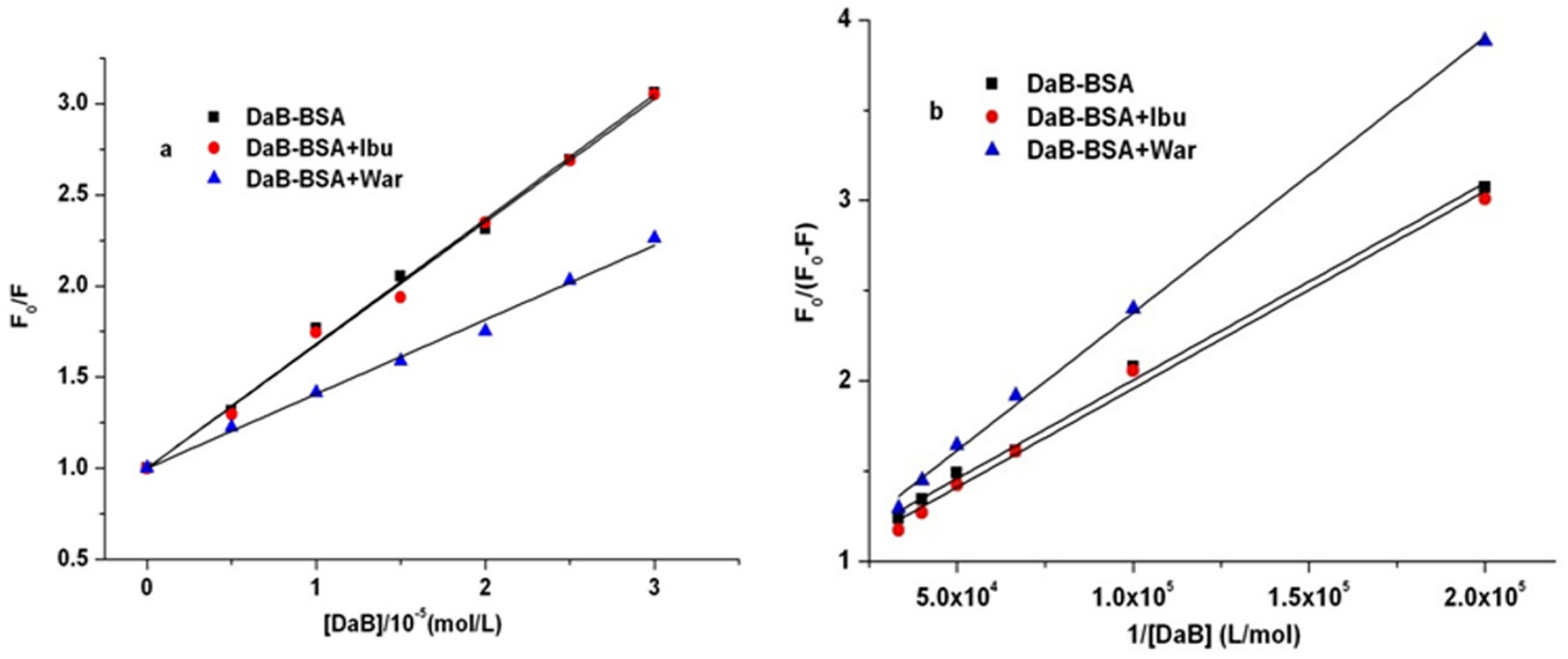
Results for Site marker competitive experiments.

In site marker competitive experiments, BSA and relative site marker were mixed in a ratio of 1:1, then different concentrations of SaB solution were prepared. The fluorescence quenching data of the SaB-BSA system in the presence of site markers were analyzed using the Eq (1) and Eq (2) as shown in Fig 8. The binding parameters were calculated and are summarized in Table 2. The binding parameters of the SaB-BSA system with warfarin decreased almost 40% compared to those of without warfarin. On the other hand, the binding parameters of the SaB-BSA system with and without ibuprofen were comparable. This result suggested that SaB bound on BSA within Sudlow's site I position.

**Table 2:** Binding parameters of competitive experiments of SaB-BSA system, at 310 K, pH 7.2.

	K_SV_ (×10^4^ M^-1^)	Kq (×10^12^ M^-1^s^-1^)	K_a_ (×10^4^ M^-1^)
SaB-BSA	6.83	6.83	8.34
SaB-BSA+ Warfarin	4.07	4.07	5.58
SaB-BSA+ Ibuprofen	6.75	6.75	7.91

### 3.4. Fluorescence studies of the SaB-BSA system coexisting with other components in DHI

In order to discuss the effects of the coexisting components from Honghua (Flos Carthami Tinctorii) in DHI on the SaB-BSA binding system, we carried out series fluorescence spectrometry studies of the SaB-BSA binding system in the presence of those components. First, BSA was mixed with the coexisting components in a 1:1 ratio. Test solutions containing different concentrations of SaB (from 0 M to 40×10^-6^ M) were prepared with fixed BSA and coexisting components at a concentration of 5×10^-6^ M.

**Fig 9:**
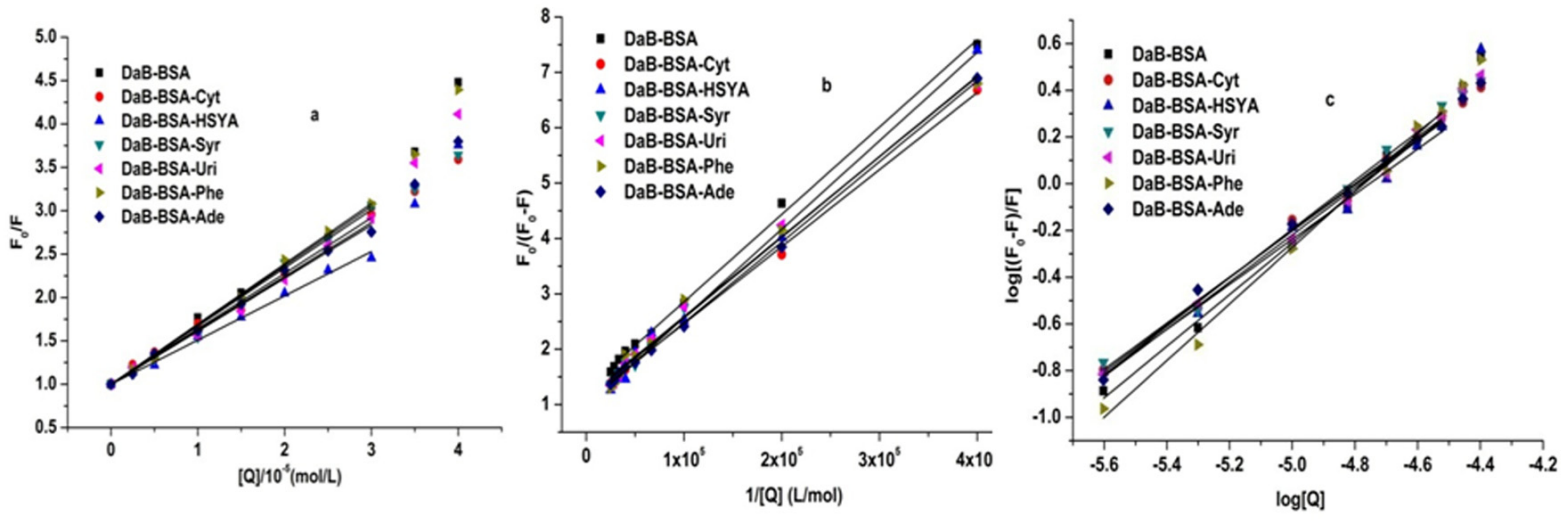
Fluorescence studies of the SaB-BSA system coexisting with other components in DHI.

As shown in Fig 9 and Table 3, SaB also caused the intrinsic fluorescence quenching of BSA in the presence of HYSA, Syr, Phe, Uri, Cyt, and Ade. In the presence of these natural components, there were still upward curvatures when the concentration was larger than 30×10^-6^ M, and within the linear concentration (from 0 M to 30×10^-6^ M), the values of K_q_ for the SaB-BSA system were still larger than 2×10^10^ M^-1^ s^-1^. These results suggested that the coexistence did not change the quenching mechanism of the SaB-BSA binding system. As shown in Table 3, HYSA, Syr, Uri, Cyt, and Ade decreased the binding parameters K_b_ of the SaB-BSA system by 21–58%, while Phe enhanced the binding parameter K_b_ of the SaB-BSA system. The changes in the other binding parameters, such as K_q,_ K_sv_, and K_a_, were similar to those of K_b_. Therefore, the participation of these natural components may affect the interaction of the SaB-BSA system.

**Table 3:** Binding parameters of SaB-BSA system in the presence of coexisted components, at 310 K, pH 7.2.

	HYSA	Syr	Phe	Uri	Cyt	Ade
K_SV_ (×10^4^ M^-1^)	5.08	6.60	6.94	6.21	6.11	6.41
K_q_ (×10^12^ M^-1^s^-1^)	5.08	6.60	6.94	6.21	6.11	6.41
K_a_ (×10^4^ M^-1^)	6.29	8.12	8.93	7.57	7.10	7.71
K’_b_ (×10^4^ M^-1^)	3.89	10.7	61.6	6.61	5.25	7.08
K’_b_/K_b_	0.21	0.58	3.31	0.36	0.28	0.38

K’_b_ is the K_b_ of SaB-BSA system in the presence of coexisted components.

**Fig 10:**
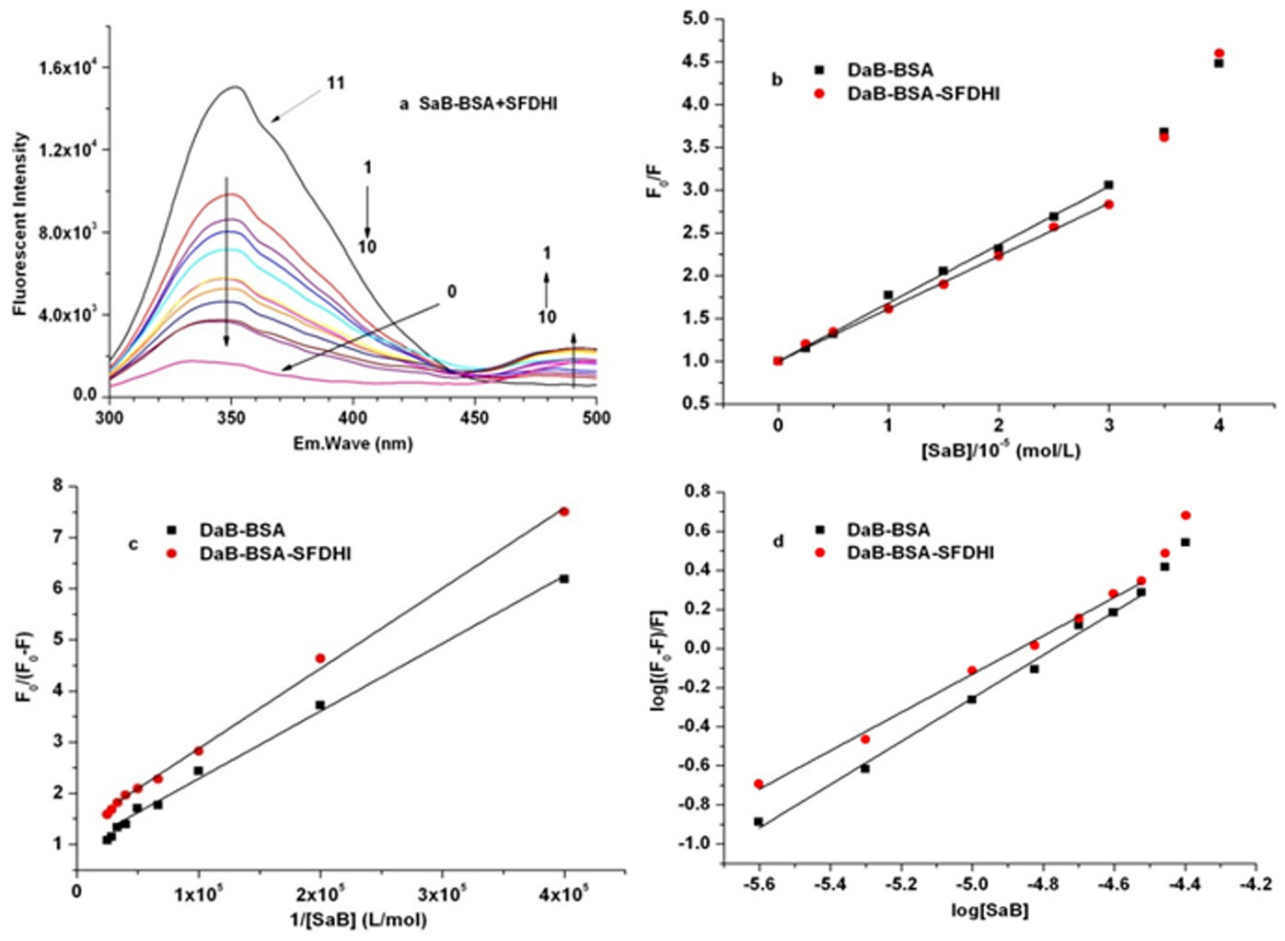
Fluorescence studies of the SaB-BSA system coexisting with other components in SFDHI.

Furthermore, to verify the interaction between SaB and BSA in the entire DHI system, our collaborator prepared special samples of DHI that specifically knocked-out SaB [45, 46]. In this experiment, the concentrations of BSA and SFDHI were 5×10^-6^ M and 0.1 gL^-1^, respectively. The concentrations of SaB varied from 0 M to 40×10^-6^ M. As shown in Fig 10 and Table 4, the fluorescence emission spectra of BSA with different concentrations of SaB in the presence of SFDHI are very similar to those in the absence of SFDHI. At the same concentrations (from 0 M to 30×10^-6^ M), the curve of F_0_/F versus [Q] was linear. When the concentration was greater than 30×10^-6^ M, the Stern-Volmer plot changed to an upward curvature, indicating a combined quenching at a higher concentration ratio of C_SaB_/C_BSA_. The value of K_q_ for SaB was still greater than 2×10^10^ M^-1^ s^-1^, indicating that the quenching also corresponded to a static quenching mechanism. The other parameters were also calculated within the linear concentration and are summarized in Table 5. Compared with the binding parameter K_b_ of “pure” SaB-BSA system, SFDHI decreased the binding parameter K_b_ of the SaB-BSA system by 32%, and the changes in the other binding parameters, such as K_q,_ K_sv_, and K_a_, agreed with those of K_b_. Based on the above studies, when DHI was used in the mammal bodies, SaB is released from serum albumin more quickly than when it is used alone, which may increase the concentrations of unbound SaB in plasma and improve the pharmacological effects of SaB.

**Table 4:** Binding parameters of SaB-BSA system in the absence and presence of SFDHI, at 310 K, pH 7.2.

	K_SV_ (×10^4^ M^-1^)	Kq (×10^12^ M^-1^s^-1^)	K_a_ (×10^4^ M^-1^)	K’_b_ (×10^4^ M^-1^)	K’_b_/K_b_
SaB-BSA	6.83	6.83	8.34	18.6	—
SaB-BSA+ SFDHI	6.16	6.16	7.33	5.89	0.32

K’_b_ is the K_b_ of SaB-BSA system in the presence of coexisted components.

**Table 5:** Docking scores for all of the natural components in DHI binding to BSA and HSA.

Compound	For HSA (Kcal/mol)		For BSA (Kcal/mol)	
	Site I	Site II	Site I	Site II
SaB	-9.7	>0	-8.3	-4.4
HSYA	-8.5	>0	-7.5	-2.3
Syr	-6.7	-5.7	-6.8	-5.6
Ade	-6.1	-6	-6.1	-5.8
Phe	-6	-6.1	-6.2	5.1
DSS	-6	-6.5	-5.8	6.1
Uri	-5.5	-6.3	-6.1	5.8
Cyt	-5.5	-6.3	-5.8	6.2

### 3.5. Molecular docking studies

AutoDock Vina-based molecular docking includes an entire surface search of the selected protein region to locatethe most optimum binding sites and an energy match calculation to identify the conformations of the ligand molecules [47–49]. The aim of this study was to evaluate the binding affinities of these components in DHI on plasma protein and to determine the most optimum binding site.

Two crystallographic structures of HSA, which co-crystallized with warfarin (entry code 1H9Z) [29] and with ibuprofen (entry code 2BXG) [30], in Protein Data Bank (PDB) was selected for docking studies. It’s because that warfarin and ibuprofen has been demonstrated to be the site marker of site I and site II. The conformation of the protein with site marker is a good starting model for further docking studies. On the other hand, the structural information of BSA with a proper site marker ligand (warfarin nor ibuprofen) was not provided in the Protein Data Bank. We chose the crystallographic structure encoded 4JK4 [28] for docking studies towards BSA. Because 4JK4 is the only crystallographic structure which can provide informations of the amio acid residues lying in site I binding pocket in the presence of a ligand. Moreover, the co-crystallized ligand of 4JK4 is located in both site I and site II binding pocket. It can also used to simulate the amio acid residues lying in site II binding pocket. The docking scores are presented in Table 5.

From the molecular docking data, we could confer that Ade, Phe, DSS, Uri, and Cyt bound very weakly to BSA and HSA, while HSYA and Syr had much higher binding ability to BSA and HSA, SaB represented the highest binding ability to both BSA and HSA. Meanwhile, the binding site of SaB, HSYA, and Syr was within Sudlow's site I. All of the docking results offered a rational molecular explanation for the previous experimental findings.

**Fig 11:**
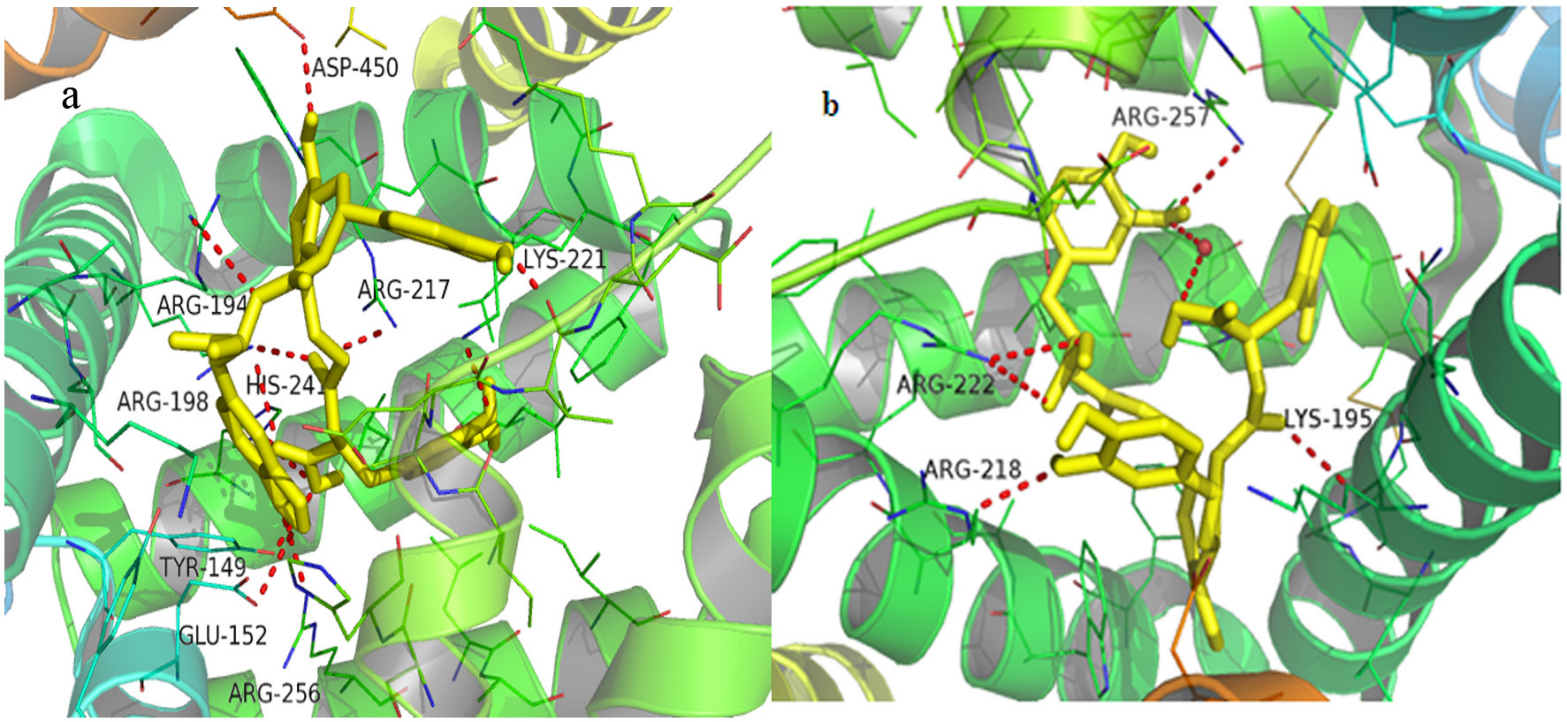
Docking orienations of SaB for binding on BSAand HSA.

It is evident that several amino acids with hydrophobic and hydrophilic characteristics were in contact with SaB in these BSA and HSA complexes and, that hydrogen bonds play an important role in the binding system. In the SaB-BSA complexes, SaB was surrounded by Tyr-156, Tyr-340, Trp-213, Val-292, Val-342, Glu-291, Glu-293,Ala-260, Ala-290, Pro-338, Pro-446, Lys-187, Lys-294, Ser-286, Ser-191, His-287, Leu-233, and Leu-259 with a binding energy of -8.3 kcal/mol (Fig 11a). Tyr-149, Glu-152, Lys-221, His-241, Arg-194, Arg-198, Arg-217, Arg-256, and Asp-450 formed hydrogen bonds with SaB. In the SaB-HSA complex, SaB was located near Ser-192, Tyr-150, Glu-153, Ala-291, and Asp-451 with a binding energy of -9.7 kcal/mol (Fig 11b). Lys-195, Arg-218, Arg-222, and Arg-257 formed hydrogen bonds with SaB, water molecules were also likely to serve as a bridge for producing hydrogen bonds.

## Conclusions

In this study, the interaction of the natural components in DHI with BSA under physiological conditions was investigated using fluorescence spectra technology. Molecular docking studies between these components and BSA/HSA were carried out to provide a molecular explanation for the binding system. According to the results, SaB exhibited a very strong ability to quench the intrinsic fluorescence of BSA through a static quenching procedure. The binding constant (K_b_) was 18.6×10^4^ M^-1^ at 310 K. HYSA showed a much lower binding ability with binding constant (K_b_) of 1.48×10^4^ M^-1^ at 310 K. Syr could enhance the intrinsic fluorescence of BSA, and the binding constant (K) was 2.7×10^4^ at 310 K. Base on these studies, we chose SaB as a research target to investigated the influence of the multi-components coexisting in DHI on serum albumin binding. The results showed that the participation of these natural components in DHI could affect the interaction of the SaB-BSA system. Therefore, we concluded that when DHI is used in mammals, SaB would be released from serum albumin more quickly, which may increase the concentrations of unbound SaB in plasma and improve the pharmacological effects of SaB. This work provides some research basis for the rational clinical use of DHI. And also provide a new perspective to reveal the compatibility principle of DHI.
